# Quality and reliability of diabetic nephropathy–related videos on TikTok and Bilibili: A cross-sectional content analysis

**DOI:** 10.1097/MD.0000000000049308

**Published:** 2026-06-12

**Authors:** Min Wang, Xinyu Wang, Baowen Gong

**Affiliations:** a Department of Nephrology, Shantou Traditional Chinese Medicine Hospital Affiliated to Guangzhou University of Chinese Medicine, Shantou, Guangdong, China; b Shantou Traditional Chinese Medicine Hospital Affiliated to Guangzhou University of Chinese Medicine, Shantou, Guangdong, China.

**Keywords:** diabetic nephropathy, health information quality, public health, social media

## Abstract

Diabetic nephropathy (DN) is a common, life-threatening complication of diabetes, contributing to the global disease burden. With the advent of video platforms, health information is being more widely disseminated. However, the quality of such content varies widely, which may influence the public’s perception. This study aimed to evaluate the upload sources, content, and characteristics of DN-related videos on TikTok and Bilibili and to explore descriptive associations between video quality scores and selected video characteristics. This cross-sectional content analysis included 166 DN-related videos. Video quality was assessed using the Global Quality Scale (GQS), modified DISCERN (mDISCERN), and Journal of the American Medical Association (JAMA) benchmark criteria. Descriptive subgroup and correlation analyses were performed to examine cross-sectional associations between video quality scores and selected video attributes. No multivariable adjustment was performed. In unadjusted cross-sectional comparisons, TikTok videos showed higher observed engagement counts at the time of data collection than Bilibili videos, whereas no statistically significant differences were observed in video duration or quality indicators after correction for multiple comparisons. In unadjusted descriptive subgroup comparisons, videos uploaded by experts showed more favorable results in selected quality-related measures, particularly GQS and JAMA, than videos uploaded by individual users. No clear association was observed between video quality and snapshot engagement metrics recorded at the time of retrieval. This study identified descriptive differences in the presentation and dissemination patterns of DN-related health information across TikTok and Bilibili. Because the analyses were observational, cross-sectional, and unadjusted for potential confounders such as video length and content type, the observed differences between platforms and uploader types should be interpreted as descriptive associations only rather than independent effects.

## 1. Introduction

Diabetic nephropathy (DN) is one of diabetes’ most prevalent and serious consequences, and it is the major cause of chronic kidney disease and end-stage kidney disease (ESKD).^[1]^ According to the Global Burden of Disease Study 2021, the global number of prevalent cases of DN was 107.6 million in 2021, corresponding to an age-standardized prevalence rate of 1259.6 per 100,000 population. In the same year, the global number of deaths from DN was 477.3 thousand, with an age-standardized mortality rate of 5.7 per 100,000 population, representing a 37.8% increase since 1990. The number of disability-adjusted life years (DALYs) attributable to DN was 11,278.9 thousand, with an age-standardized DALY rate of 131.1 per 100,000 population, reflecting a 24.0% increase since 1990. These data show that during the last 3 decades, both mortality and DALY rates for DN have continuously climbed worldwide, making it a major public health concern.^[2]^ DN is not only a leading cause of ESKD and kidney failure but also significantly increases the risk of cardiovascular diseases such as hypertension, arteriosclerosis, and heart disease.^[3]^ Many DN patients need long-term dialysis therapy in the later stages. This further reduces their quality of life and places a significant financial strain on them.^[4]^Furthermore, the loss of renal function causes many patients to progressively lose their capacity to take care of themselves, develop severe disabilities, and be unable to return to normal social functioning or work capacity, all of which severely restrict their ability to participate in social activities and realize their own worth.^[5]^The disease’s detrimental effects on quality of life are further exacerbated by severe complications, persistent physical discomfort, and frequent dialysis procedures, which all lead to the slow establishment of emotional disorders including anxiety and depression.^[6]^ DN often has a subtle onset. Surveys show that around half of individuals develop microalbuminuria when they are first diagnosed with diabetes. The progression to ESKD eventually results from a decline in the glomerular filtration rate at a rate of 2–20 mL/min annually after persistent (overt) proteinuria occurs.^[7]^ In order to slow the progression of the condition, early screening, lifestyle changes, and stringent blood glucose, blood pressure, and blood lipid control are essential.^[8]^ However, many patients may miss the early “reversible window period” and hence not receive appropriate screening or intervention due to the lack of public knowledge about DN.

The dissemination of health information has changed as a result of the digital revolution. Online video platforms have emerged as one of the most popular ways for Chinese people to learn about health and medicine in recent years. Compared to searching for and comprehending lengthy traditional academic literature dominated by specialized terminology, video platforms such as TikTok and Bilibili significantly reduce barriers for the general public to understand medical and health knowledge through features such as videos, real-time comment interactions, and algorithmic recommendations.^[9]^ However, the decentralized production paradigm impairs quality control procedures, resulting in variable video quality. Using diabetes as an example, research has found that <20% of diabetes-related videos on YouTube meet evidence-based medical standards, and there is a negative link between the number of views and the quality of these films.^[10]^ Such inaccurate information not only wastes medical resources, but it may also induce patients to abandon medicine or follow incorrect diets and treatments, causing considerable harm to their health.^[11]^

TikTok and Bilibili were selected because they are 2 major video-sharing platforms in China with different content dissemination styles and user interaction patterns, making them suitable for a cross-platform comparison of DN-related health information. Although previous studies have examined the quality of TikTok videos on topics such as cervical cancer and COVID-19 vaccinations, no research has yet focused on DN. Given that the prevention and management of DN require long-term self-management and early behavioral interventions, it is crucial to assess the credibility and scientific accuracy of DN-related content on major short video platforms to improve online health communication governance and enhance public health literacy. In this context, the present study aims to explore several aspects of DN-related video content on TikTok and Bilibili. Specifically, this study addresses the following primary research questions: What is the overall quality and credibility of DN-related short videos on TikTok and Bilibili? Are there descriptive differences in content quality and communication characteristics between the 2 platforms? And do these descriptive differences vary according to the type of content creator? In order to investigate these questions, this study will conduct a thorough assessment of DN-related short films on TikTok and Bilibili, analyzing the quality, credibility, and distribution features of the videos. It will also compare descriptive differences between platforms and types of content creators, providing descriptive evidence that may inform the future development of high-quality and trustworthy digital educational materials on DN. Ultimately, the study aims to contribute to a better understanding of how DN-related information is presented online, which may help inform future efforts to improve public awareness and disease self-management education.

## 2. Methods

### 2.1. Ethics approval

This study analyzed only publicly available videos from TikTok and Bilibili. It did not involve human participants, clinical data, laboratory animals, histological materials, access to private information, or any interaction with users. No personally identifiable information was collected or processed. Therefore, institutional ethics committee approval and informed consent were not required.

### 2.2. Platform selection

TikTok and Bilibili were purposively selected because they are 2 of the most influential video-based social media platforms in China and represent distinct but complementary modes of online health communication. TikTok is characterized by short-form, algorithm-driven, high-velocity dissemination, whereas Bilibili more often supports longer, topic-oriented, and relatively in-depth video presentations. Prior studies have shown that these platforms are both important channels for the dissemination of health information in China, while also differing in video duration, interaction patterns, creator ecology, and recommendation mechanisms, making them suitable for comparative analysis of online medical content quality and communication characteristics.^[12,13]^ The methodological rationale for selecting these platforms was also informed by previous cross-sectional studies of health-related videos on social media, which commonly compare platforms with different communication logics to evaluate variations in content quality, reliability, and user engagement.^[12,14,15]^ Therefore, TikTok and Bilibili were chosen because they are 2 representative Chinese video platforms with different content formats, recommendation logics, and communication features, making them appropriate for examining how DN-related health information is presented across distinct online video environments.

### 2.3. Search strategy and inclusion/exclusion criteria

To ensure the data is thorough and representative, we utilized the Chinese term “糖尿病肾病” (diabetic nephropathy) as the search keyword to collect data separately on the TikTok and Bilibili platforms. The search and screening process was designed with reference to previous cross-sectional studies evaluating health-related video content on social media platforms.^[12–15]^ To reduce bias caused by platform-specific personalized recommendations, searches were performed using newly registered accounts in incognito mode, ensuring that neither browsing history nor personalized algorithms influenced the search results. After the searches, the top 100 videos from each platform were selected using the default sorting mechanism (i.e., without tailored filtering) for further screening.

Given the dynamic nature of platform content, where videos may be modified, deleted, or made private over time, all searches were completed within the same study period to reduce temporal variation in content availability. Videos that were no longer accessible during the screening process, including those that had been deleted, privatized, or linked to broken pages, were excluded from the analysis. Only videos that remained available at the time of eligibility confirmation were included in the final sample. Because this was a cross-sectional content analysis, no repeated follow-up of video availability was performed after data extraction.

To enhance reproducibility and consistency, we recorded each video’s URL and metadata at the time of retrieval, including source platform, uploader type, video length, likes, comments, shares, and collections. Engagement metrics were captured as raw counts at a single time point and were therefore treated as cross-sectional snapshots rather than stable or time-standardized indicators of audience response. Because upload dates and comparable time-based engagement measures were not consistently available across platforms, temporal standardization of engagement metrics was not performed.

### 2.4. Data extraction

Data extraction included the following: video features include platform (TikTok or Bilibili), uploader type (expert, nonexpert, or individual user), video duration (seconds), and URL. Engagement indicators included the number of likes, comments, shares, and favorites recorded at the time of retrieval. These variables were treated as cross-sectional snapshot measures of engagement rather than temporally standardized indicators. Content categories include 6 topics: diagnosis, epidemiology, causation, prevention, symptoms, and treatment. Quality assessment: video quality was evaluated using several widely accepted tools, including the Global Quality Score (GQS), the modified DISCERN (mDISCERN), and the Journal of the American Medical Association (JAMA) benchmark criteria.

All data were extracted independently by 2 medical researchers. Before the formal evaluation, both researchers underwent training to ensure a consistent understanding and application of the assessment criteria, thereby reducing subjective bias. Interrater reliability was assessed using linear weighted Cohen kappa (κ) based on the initial independent ratings. Any discrepancies between the 2 reviewers were resolved by a senior specialist in diabetic nephropathy. The final extracted data were entered into Excel (Microsoft Corp). The final coding sheet and scoring form are provided (see Table, Supplemental Digital Content, which illustrates the coding sheet of video variables).

### 2.5. Video quality assessment

To analyze the quality and reliability of short videos, we used 3 assessment tools: GQS, the mDISCERN score, and the JAMA benchmark criteria. Each of these tools was chosen for its ability to evaluate different aspects of video quality, allowing for a comprehensive assessment. The GQS employs a 5-point Likert scale (1 = poor, 5 = excellent) to provide an overall assessment of each video’s professionalism, comprehensiveness of material, clarity of presentation, and viewer comprehension. This tool offers a broad evaluation of the video’s overall quality and effectiveness in conveying medical information (Table 1).^[16]^ The mDISCERN instrument, based on the original DISCERN tool, has 5 binary items: clarity, relevance, traceability, robustness, and fairness. Each item that meets the requirement receives 1 point, giving a total score ranging from 0 to 5. Higher values indicate greater reliability and quality of health information, specifically evaluating how well the content holds up in terms of accuracy and trustworthiness (Table 2).^[17]^ The JAMA score is based on 4 transparency criteria set by the Journal of the American Medical Association: authorship, credit, references, and disclosure. Each criterion met receives 1 point, giving a total score of 0 to 4. This score is designed to assess the transparency and credibility of the video’s content (Table 3).^[18]^ These 3 tools were used in combination to provide a comprehensive evaluation of the video content from different perspectives: GQS focuses on overall presentation and professionalism, mDISCERN evaluates the reliability and robustness of the information, and JAMA assesses the transparency and credibility of the sources. This multi-faceted approach ensures a thorough and balanced assessment of video quality.

**Table 1:** Description of the Global Quality Score (GQS) 5-point scale used to evaluate videos with diabetic nephropathy information.

GQS	Description
1	Poor quality, poor flow of the site; most information missing; not at all useful for patients.
2	Generally poor quality and poor flow, some information listed but many important topics missing, of very limited use to patients.
3	Moderate quality, suboptimal flow, some important information is adequately discussed but others poorly discussed: somewhatuseful for patients.
4	Good quality and generally good flow, most of the relevant information is listed, but some topics not covered, useful for patients.
5	Excellent quality and excellent flow; very useful for patients

**Table 2:** Modified DISCERN quality criteria for assessing the reliability of video. (1 point for answer “yes”, 0 point for nswer “no”.

Item number	Reliability score
1	Is the video clear, concise, and understandable?
2	Are valid sources cited?
3	Is the content presented balanced and unbiased?
4	Are additional sources of content listed for patient reference?
5	Are areas of uncertainty mentioned?

**Table 3:** The Journal of American Medical Association (JAMA) benchmark criteria.

Criteria	Description
**Authorship**	Authors and contributors, their affliations, and relevant credentials should be provided.
**Attribution**	References and sources for all content should be listed clearly, and all relevant copyright information noted.
**Currency**	Website ownership should be prominently and fully disclosed, as should any sponsorship, advertising, underwriting, commercialfunding arrangements or support, or potential.
**Disclosure**	Dates that content was posted and updated should be indicated.

### 2.6. Statistical analysis

Descriptive statistical approaches were utilized to summarize both continuous and categorical variables. Continuous variables with normal distributions were presented as mean ± standard deviation, while nonnormal distributions were expressed as median and interquartile range (IQR). Categorical variables were reported using frequencies and percentages. For group comparisons, continuous variables with a normal distribution were analyzed using the independent-samples *t* test, whereas non-normally distributed variables were studied using the Mann–Whitney *U* test. The Kruskal-Wallis *H* test was used to compare 3 or more sets of data. When the Kruskal-Wallis test gave significant findings, the Dunn post hoc test was used for pairwise comparisons to determine which groups were different. In addition, to reduce the risk of type I error arising from multiple statistical comparisons, Bonferroni correction was applied where appropriate by multiplying the original *P*-values by the number of comparisons performed (with adjusted *P*-values capped at 1.00), and the adjusted *P*-values were used to determine statistical significance. Finally, the Spearman rank correlation coefficient was used to assess the relationship between video quality scores and snapshot engagement metrics recorded at the time of retrieval. No multivariable regression or other adjusted analysis was performed. Therefore, all between-platform and between-uploader comparisons should be interpreted as exploratory, unadjusted descriptive comparisons rather than independent associations. Potential confounding by video duration, content type, and other unmeasured factors cannot be excluded. All statistical tests were 2-sided, and a *P*-value of <0.05 was considered statistically significant. All statistical analyses were carried out using R (version 4.3.2).

## 3. Results

### 3.1. Overall characteristics of videos

**Figure 1.**
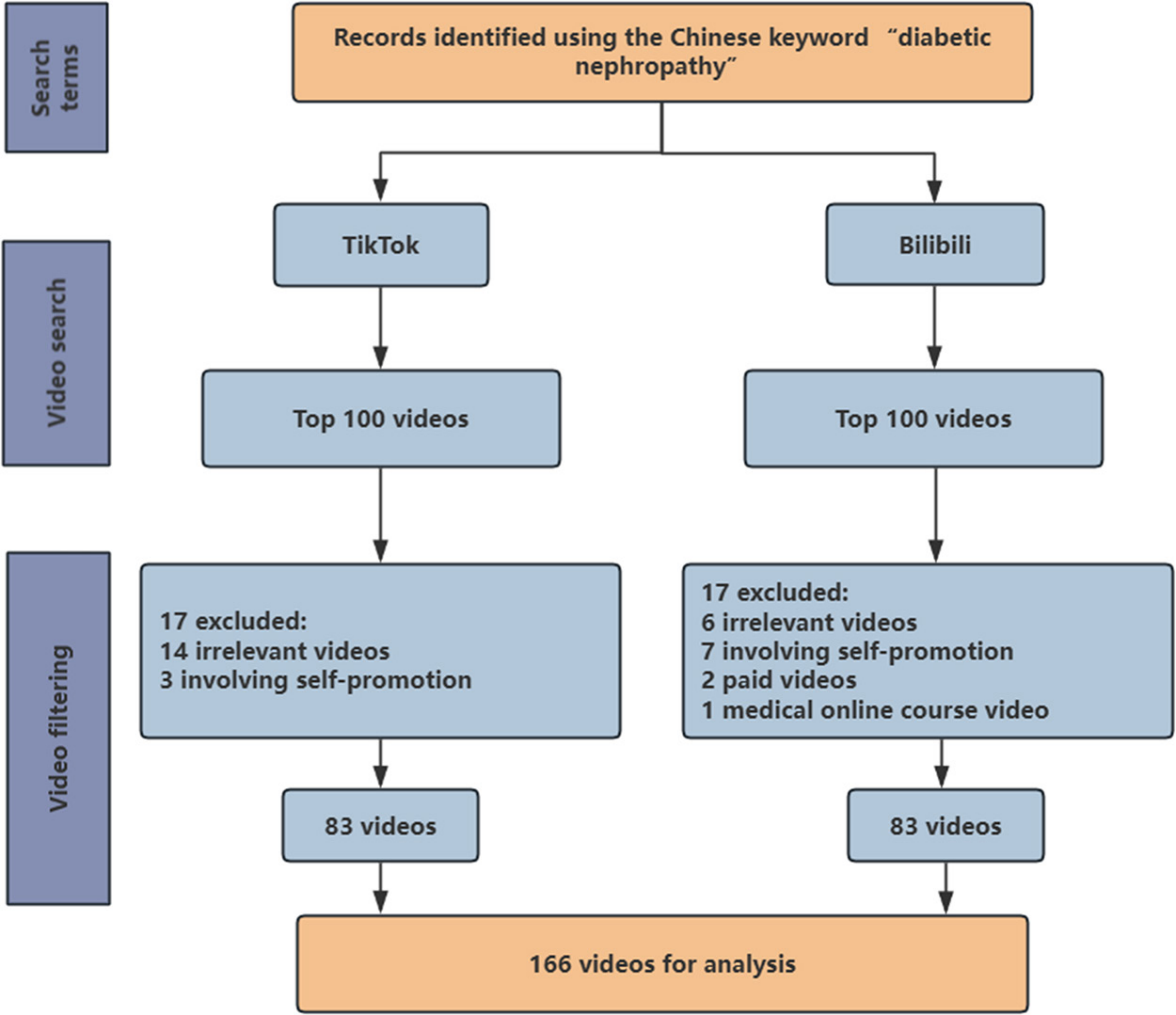
Flowchart for searching and selecting videos on diabetic nephropathy.

**Figure 2.**
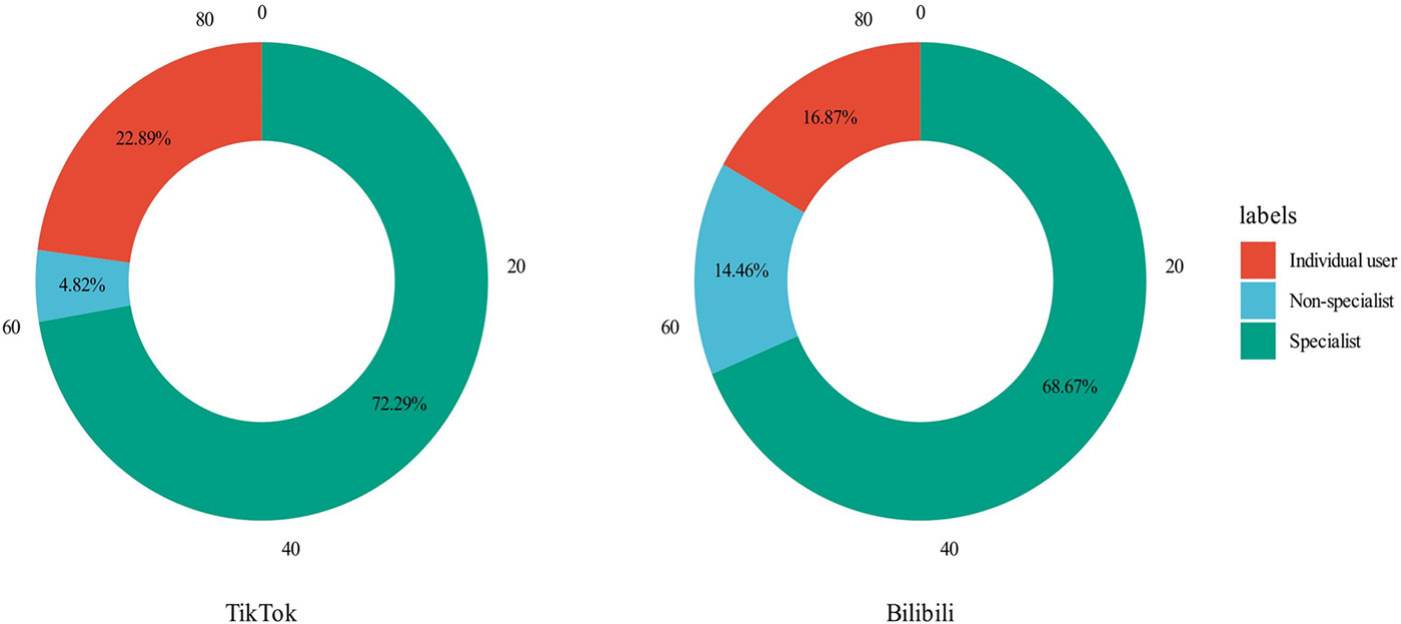
Proportions of diabetic nephropathy-related videos uploaded to TikTok and Bilibili.

We gathered the top 100 videos from TikTok and Bilibili. After applying the exclusion criteria, the final sample consisted of 166 videos. (Fig. 1) illustrates the detailed screening process. (Table 4) shows the features of the videos that were included.In terms of platform distribution, TikTok and Bilibili contributed 83 videos apiece, accounting for 50%. Experts uploaded the most videos (74.1%), followed by nonexperts (10.2%) and ordinary users (15.7%). (Fig. 2) illustrates the distribution of video makers across various platforms. On TikTok, videos published by experts accounted for 72.29%, nonexpert videos for 4.82%, and individual user videos for 22.89%. Expert-uploaded videos accounted for 68.67% of all videos on Bilibili, followed by nonexpert videos (14.46%) and individual user videos (16.87%). The median number of likes for all included videos was 118.5 (IQR: 9.00–857.50); the median number of comments was 9.00 (IQR: 0.00–62.25); the median number of favorites was 57.00 (IQR: 9.00–496.00), and the median number of shares was 26.00 (IQR: 3.00–199.00). The median video duration was 120.5 seconds (IQR: 65.25–213.50).In terms of video quality, the median GQS score was 3.00 (IQR: 3.00–3.00), the median mDISCERN score was 3.00 (IQR: 2.00–3.00), and the median JAMA score was 2.00 (IQR: 2.00–2.00).

**Table 4:** Video characteristics.

Variables	Value
Number of likes [median (IQRa)]	118.50 (9.00, 857.50)
Number of comments [median (IQR)]	9.00 (0.00, 62.25)
Number of collections [median (IQR)]	57.00 (9.00, 496.00)
Number of shares [median (IQR)]	26.00 (3.00, 199.00)
Video length [s, median (IQR)]	120.50 (65.25, 213.50)
GQSb scores [median (IQR)]	3.00 (3.00, 3.00)
mDISCERNc scores [median (IQR)]	3.00 (2.00, 3.00)
JAMAd scores [median (IQR)]	2.00 (2.00, 2.00)

GQS = Global Quality Score, IQR = interquartile range, JAMA = Journal of American Medical Association, mDISCERN = modified Decision-making Information Support Criteria for Evaluating the Reliability of Non-randomized Studies.

### 3.2. Distribution of video content

**Figure 3.**
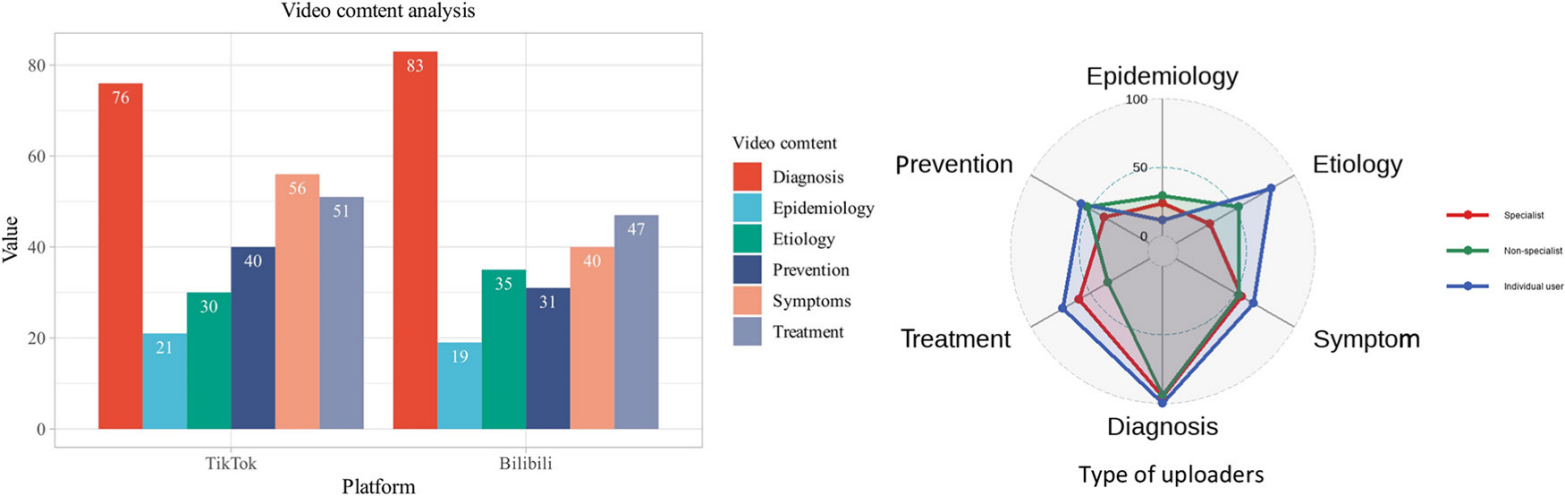
Distribution of attention across 6 diabetic nephropathy-related themes by platform and uploader type.

(Fig. 3) depicts how different platforms and uploaders prioritize 6 components of DN (diagnosis, epidemiology, etiology, prevention, symptoms, and treatment). On the TikTok platform, videos relating to diagnosis received the most views (72.29%), followed by symptoms and treatment (67.47% and 61.45%, respectively). Content concerning prevention, etiology, and epidemiology was less common, accounting for 48.19%, 36.14%, and 25.30%, respectively. On the Bilibili platform, diagnosis accounted for 100% of all videos, with treatment and symptom-related content accounting for 56.63% and 48.19%, respectively. Similar to TikTok, conversations on prevention, etiology, and epidemiology were sparse on Bilibili, with 37.35%, 42.17%, and 22.89%, respectively. Furthermore, the 3 types of uploaders: experts, nonexperts, and individual users, focused mostly on diagnosis, symptoms, and treatment, with relatively little attention paid to prevention, etiology, and epidemiology.

### 3.3. Unadjusted descriptive comparison of video duration, engagement, and quality between TikTok and Bilibili

**Figure 4.**
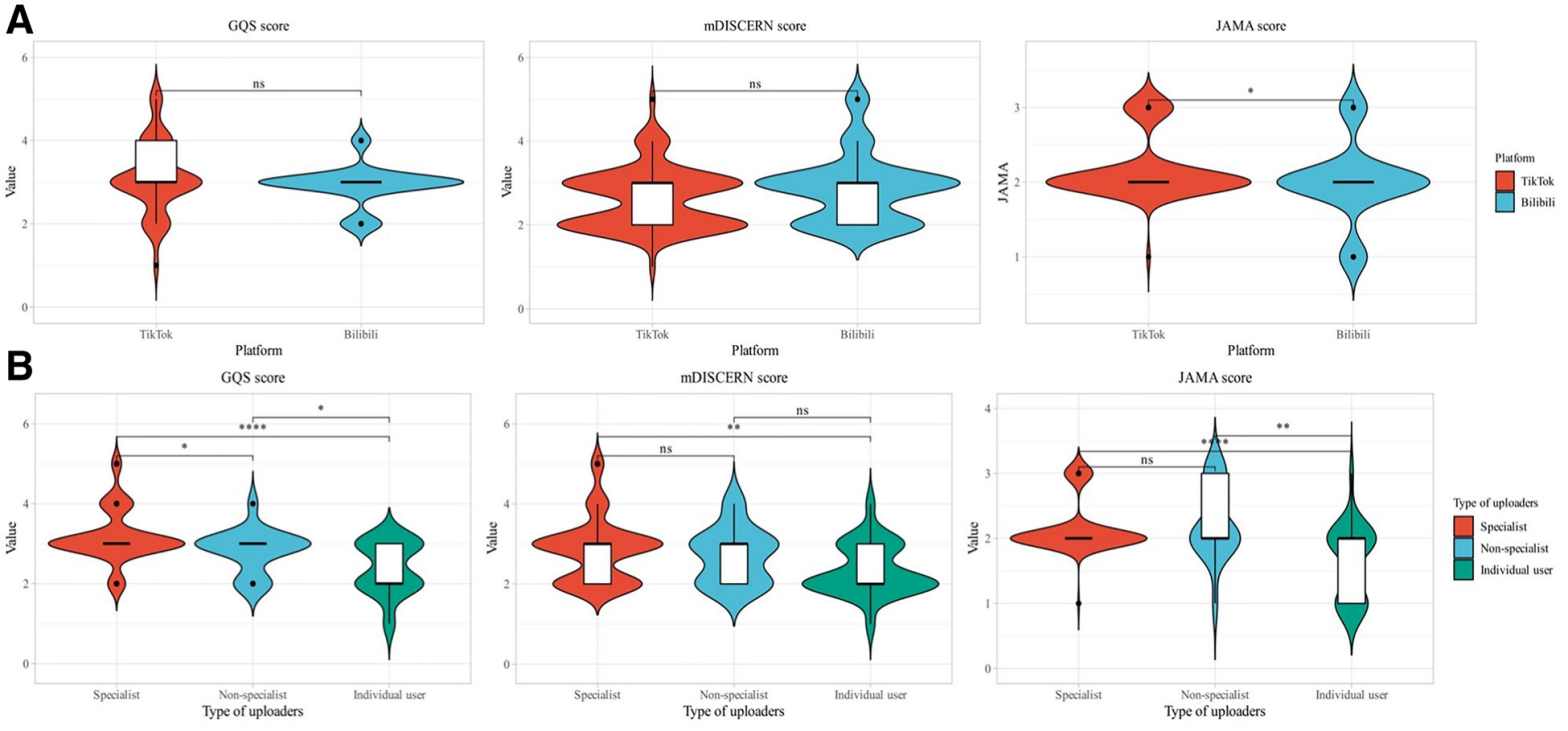
(A)Violin plots comparing scores from 3 assessment tools between TikTok and Bilibili. (B)Violin plots comparing scores from 3 assessment tools across different uploader types.

We conducted an unadjusted descriptive comparison of TikTok and Bilibili in terms of video duration, engagement, and quality (Table 5). In terms of video length, TikTok videos had a median duration of 117 seconds (IQR: 50.5–162.5 seconds), whereas Bilibili videos had a median duration of 124 seconds (IQR: 72–349.5 seconds). Although the raw comparison suggested a difference between the 2 platforms (*P* = .029), this difference was not statistically significant after Bonferroni correction. TikTok videos had higher observed engagement counts at the time of data collection than Bilibili videos, including likes, comments, favorites, and shares (all adjusted *P*-values < 0.001). Specifically, the median number of likes was 863 (IQR: 288–4779) on TikTok versus 9 (IQR: 2–29.5) on Bilibili; the median number of comments was 51 (IQR: 18.5–303) versus 0 (IQR: 0–2.5); the median number of favorites was 419 (IQR: 99.5–1554.5) versus 10 (IQR: 1.5–50); and the median number of shares was 156 (IQR: 48.5–1494) versus 3 (IQR: 0–17), respectively. In contrast, no statistically significant between-platform differences were observed in GQS, mDISCERN, or JAMA scores after Bonferroni correction (adjusted *P* = .680, 0.448, and 0.152, respectively). Overall, in this unadjusted cross-sectional comparison, the 2 platforms differed in observed engagement counts at the time of retrieval, whereas no statistically significant differences were observed in video duration or any of the 3 quality indicators after correction for multiple comparisons. These between-platform findings should be interpreted as exploratory, unadjusted cross-sectional observations rather than independent platform effects (Fig. 4a). Interobserver reliability was assessed using linear weighted Cohen kappa, with values of 0.81 (95% CI: 0.73–0.88) for GQS, 0.89 (95% CI: 0.84–0.94) for mDISCERN, and 0.83 (95% CI: 0.72–0.91) for JAMA.

**Table 5:** Comparison of characteristics between TikTok and Bilibili.

Variables	Bilibili (N = 85)	TikTok (N = 83)	Statistic	*P*-value (raw)	*P*-value (bonferroni corrected)
Video length (s, median [IQR])	124.00 (72.00, 349.50)	117.00 (50.50, 162.50)	*Z* = −2.18	.029	.232
Number of likes (median [IQR])	9.00 (2.00, 29.50)	863.00 (288.00, 4779.00)	*Z* = −10.45	<.001	<.001
Number of comments (median [IQR])	0.00 (0.00, 2.50)	51.00 (18.50, 303.00)	*Z* = −9.64	<.001	<.001
Number of collections (median [IQR])	10.00 (1.50, 50.00)	419.00 (99.50, 1554.50)	*Z* = −8.71	<.001	<.001
Number of shares [median (IQR)]	3.00 (0.00, 17.00)	156.00 (48.50, 1494.00)	*Z* = −8.76	<.001	<.001
GQS scores (median [IQR])	3.00 (2.00, 3.00)	3.00 (3.00, 4.00)	*Z* = −1.72	.085	.680
mDISCERN scores [median (IQR)]	3.00 (2.00, 3.00)	3.00 (2.00, 3.00)	*Z* = −1.87	.061	.448
JAMA (median [IQR])	2.00 (2.00, 2.00)	2.00 (2.00, 2.00)	*Z* = −2.35	0.019	.152

Mann–Whitney *U* test was used for comparisons. Raw *P*-values are shown. Adjusted *P*-values were calculated using Bonferroni correction for 8 comparisons. Adjusted *P*-values <0.05 are considered statistically significant.

### 3.4. Unadjusted descriptive comparison of video engagement, duration, and quality across different uploader types

**Figure 5.**
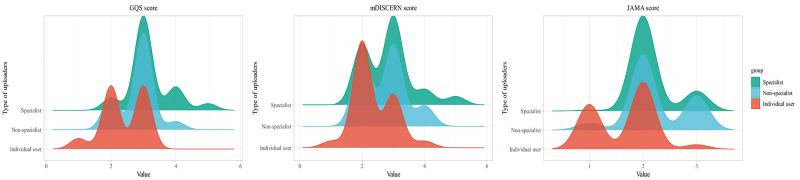
Ridge plots comparing scores from 3 assessment tools across different uploader types.

We divided the sample into 3 uploader categories: experts, nonexperts, and individual users, and conducted unadjusted descriptive comparisons of their engagement metrics, video duration, and quality (Table 6). In terms of engagement metrics, there was no significant difference in the amount of likes amongst the 3 uploader groups. Although the raw comparisons suggested disparities in comments and collections, these differences were not statistically significant after Bonferroni correction. In contrast, there was a significant difference in shares, with expert-uploaded videos showing lower sharing counts than videos uploaded by individual users and nonexperts. The median length of expert videos was 98 seconds (IQR: 50.5–155.5), significantly shorter than that of the nonexpert group (317 seconds, IQR: 132–698) and the individual user group (285.5 seconds, IQR: 115.75–674.25) (adjusted *P* < .001). The expert and nonexpert groups had higher median GQS scores (both 3.00) than the individual user group, although pairwise comparisons indicated that the clearest difference was between experts and individual users. The mDISCERN scores did not differ significantly across uploader groups after Bonferroni correction. For JAMA, the median score was 2.00 in all 3 groups, but the score distribution was more concentrated in the expert group (2.00–2.00) than in the nonexpert group (2.00–3.00) and the individual user group (1.00–2.00), and pairwise comparisons suggested differences between experts and the other 2 groups (Fig. 4b). Overall, these unadjusted descriptive subgroup comparisons suggested differences across uploader groups in video duration, sharing behavior, and selected quality-related measures, particularly GQS and JAMA. Figure 5 further shows that expert-uploaded videos tended to have more concentrated GQS, mDISCERN, and JAMA scores, whereas videos uploaded by individual users showed lower scores and greater dispersion. These subgroup findings should be interpreted as exploratory, unadjusted descriptive differences rather than independent effects of uploader type.

**Table 6:** Comparison of characteristics between different uploader types.

Variables	Individual user (n = 26)	Nonspecialists (n = 17)	Specialists (n = 123)	Statistic	Overall adjusted *P*-value	Pairwise comparisons (adjusted *P*-value)
Video length (s, median [IQR])	285.50 (115.75, 674.25)	317.00 (132.00, 698.00)	98.00 (50.50, 155.50)	*χ*^2^ = 27.63	<.001	Ind vs Non: 1.000 Ind vs Spe: <.001 Non vs Spe: < .001
Number of likes [median (IQR)]	267.00 (40.50, 5978.25)	79.00 (22.00, 641.00)	142.00 (6.00, 719.00)	*χ*^2^ = 5.80	.440	–
Number of comments [median (IQR)]	40.00 (1.00, 692.75)	3.00 (0.00, 42.00)	10.00 (0.00, 46.00)	*χ*^2^ = 7.13	0.224	–
Number of collections (median [IQR])	119.00 (37.00, 1590.00)	81.00 (51.00, 637.00)	43.00 (5.50, 305.00)	*χ*^2^ = 8.63	0.104	–
Number of shares (median [IQR])	91.50 (15.50, 3036.50)	60.00 (15.00,2 04.00)	17.00 (1.00, 136.50)	*χ*^2^ = 14.48	<.001	Ind vs Non: 1.000 Ind vs Spe: < 0.001 Non vs Spe: .040
GQS scores (median [IQR])	2.00 (2.00, 3.00)	3.00 (3.00, 3.00)	3.00 (3.0, 3.00)	*χ*^2^ = 30.11	<.001	Ind vs Non: .097 Ind vs Spe: <.001 Non vs Spe: .300
modified DISCERN scores (median [IQR])	2.00 (2.00, 3.00)	3.00 (2.00, 3.00)	3.00 (2.00, 3.00)	*χ*^2^ = 9.10#	.088	–
JAMA (median [IQR])	2.00 (1.00, 2.00)	2.00 (2.00, 3.0)	2.00 (2.00, 2.00)	*χ*^2^ = 24.25#	<.001	Ind vs Non: 1.000 Ind vs Spe: < .001 Non vs Spe: < .001

Kruskal-Wallis *H* test was used for overall comparisons. Overall adjusted *P*-values were calculated using Bonferroni correction for 8 comparisons. Pairwise adjusted *P*-values were calculated using Dunn post hoc test with Bonferroni correction for 3 comparisons. Adjusted *P*-values < 0.05 are considered statistically significant. Ind: Individual user; Non: Nonspecialist; Spe: Specialist. “—” indicates not applicable (overall *P* ≥ .05).

### 3.5. Correlation analysis between video features and quality: a comparison of TikTok and Bilibili

**Figure 6.**
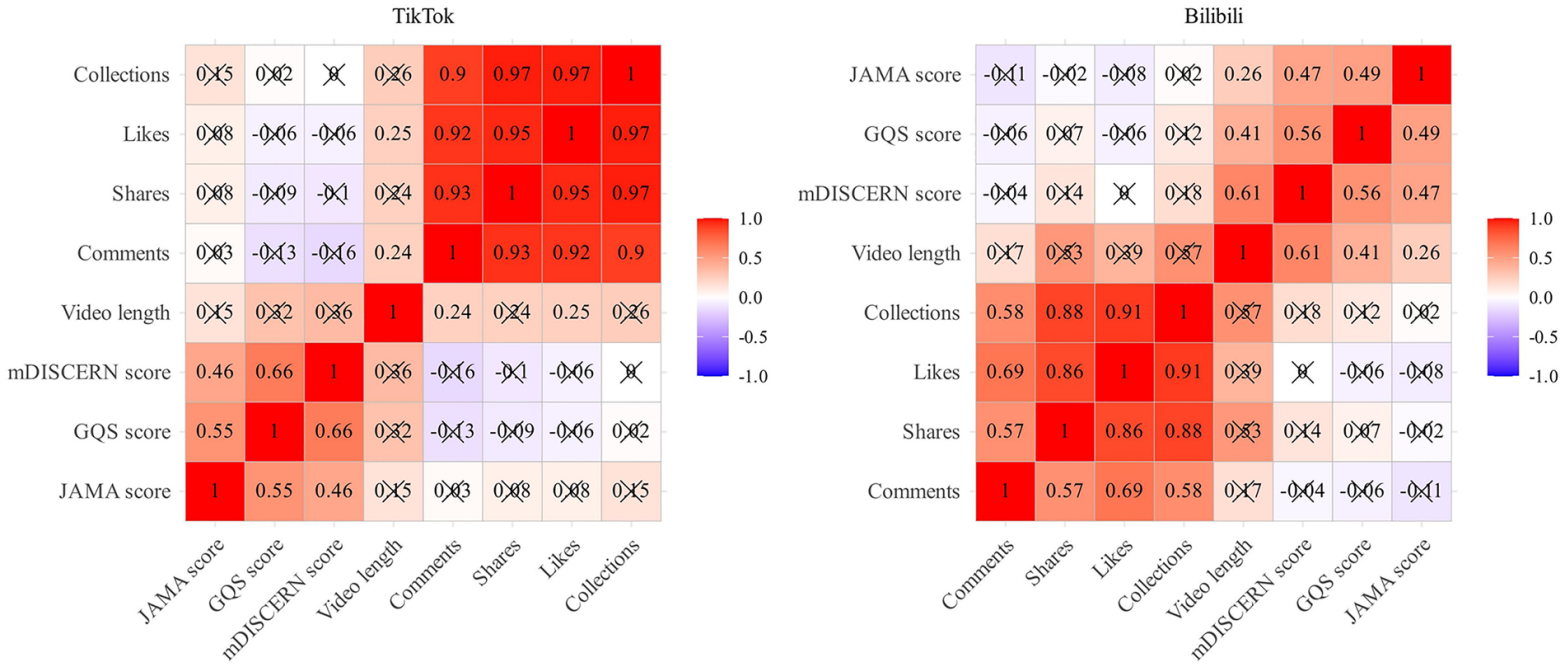
Correlation matrix of video engagement metrics and quality scores on TikTok and Bilibili.

(Fig. 6) Shows a correlation analysis of video attributes and quality scores on the TikTok and Bilibili platforms. The findings show that user engagement behaviors on TikTok were strongly interrelated. The amount of likes was strongly positively correlated with shares (*R* = 0.95) and favorites (*R* = 0.97), indicating that these snapshot engagement metrics tended to co-occur on TikTok. Furthermore, the number of comments has a strong positive correlation with the number of likes (*R* = 0.92) and shares (*R* = 0.93), indicating a high degree of interrelationship among TikTok engagement metrics. However, this does not imply that 1 sort of contact immediately benefits another. The positive associations between video length and likes (*R* = 0.25) and comments (*R* = 0.24) were weak, with no correlation observed with shares or favorites. These results suggest that video length was only weakly associated with TikTok engagement metrics. In contrast, the interaction structure on the Bilibili platform showed a different pattern. The number of likes remained substantially positively connected with shares (*R* = 0.86) and favorites (*R* = 0.91), whereas comments showed only somewhat positive correlations with likes (*R* = 0.69) and shares (*R* = 0.57). These results indicate that comments were less strongly correlated with other engagement metrics on Bilibili than on TikTok. Bilibili’s video duration had no correlation with any interaction metrics; however, it did show moderate positive correlations with the GQS and mDISCERN scores (*R* = 0.61 and 0.41, respectively), and a weak correlation with the JAMA score (*R* = 0.26). These findings suggest that, on Bilibili, longer video duration was associated with higher GQS and mDISCERN scores, whereas TikTok did not exhibit a comparable pattern. Although the video quality scores (JAMA, GQS, and mDISCERN) on both TikTok and Bilibili showed moderate positive correlations (*R* = 0.40–0.70), no correlation was observed between video quality scores and the snapshot engagement metrics recorded at the time of retrieval on either platform. These findings suggest that cross-sectional engagement measures may not directly reflect the objective quality of health information and should be interpreted cautiously.

## 4. Discussion

### 4.1. Principal results

In unadjusted cross-sectional comparisons, TikTok videos had higher observed engagement counts at the time of data retrieval than Bilibili videos, whereas no statistically significant differences were observed in video duration or quality indicators after correction for multiple comparisons. These between-platform differences should be interpreted cautiously as descriptive cross-sectional observations. Prior studies have described TikTok as emphasizing rapid short-form interaction and high viewer engagement, whereas Bilibili may support relatively more discussion-oriented and topic-focused viewing experiences.^[19,20]^ However, these platform-related explanations remain tentative because the comparisons in the present study were descriptive and unadjusted.

Unadjusted descriptive subgroup comparisons suggested differences across uploader groups in video duration and selected quality-related measures. In particular, expert-uploaded videos were shorter in duration, and the clearest between-group differences were observed for GQS and JAMA. These descriptive findings are broadly consistent with previous studies reporting that health education short videos from expert or professionally identified sources may show more favorable quality-related characteristics.^[14]^ In the present study, videos uploaded by individual users tended to show lower GQS scores and greater variability in selected quality-related measures, whereas JAMA scores were more concentrated among expert-uploaded videos. However, these findings should be interpreted cautiously because not all quality indicators differed significantly after correction for multiple comparisons and no multivariable adjustment was performed. Accordingly, the observed differences should not be interpreted as independent effects of uploader type, but as descriptive subgroup differences observed in this sample. Although this study did not assess these factors directly, previous research suggests that source transparency and professional identification may also be relevant to how online health information is perceived and evaluated by users.^[21]^

According to content analysis, the most frequently addressed themes were diagnosis and symptoms, with etiology, epidemiology, and prevention receiving less emphasis. Although discussions about diagnosis and symptoms are necessary for patients to recognize disease and seek appropriate care, limited attention to etiology, epidemiology, and disease prevention may reduce the comprehensiveness of health information available to viewers. In DN, etiological understanding and early lifestyle intervention are important for disease control and progression. Studies have shown that early lifestyle changes, such as a healthy diet, regular exercise, and weight management, may help delay the onset or progression of DN.^[22]^ The limited emphasis on etiology, epidemiology, and preventive measures on mainstream video platforms may affect viewers’ understanding of early screening and preventive strategies for DN.^[13,23]^ To improve the completeness of public health communication, platforms and content creators may consider presenting disease-related information in a more balanced manner, with greater attention to etiology, prevention, and early intervention strategies.

The correlation analysis of video attributes and quality scores demonstrated strong positive correlations among the snapshot engagement metrics on the TikTok platform, indicating that likes, comments, shares, and favorites recorded at the time of retrieval were closely interrelated.. This pattern may be partly related to the platform’s recommendation mechanism and real-time feedback architecture, although this interpretation remains speculative.^[24]^ In contrast, on the Bilibili platform, video duration was positively associated with both mDISCERN and GQS, suggesting that longer videos on this platform were associated with higher quality scores. In line with this finding, Lei et al reported a modest positive association between the duration of pancreatic cancer videos and GQS on Bilibili, with each additional minute of video duration increasing the sharing rate by 0.65 times.^[15]^ This feature contrasts with TikTok’s brief, fast-paced interaction style and may potentially reflect differences between the 2 platforms in algorithmic logic, user demographics, and content consumption habits.^[25]^ However, no correlation was observed between video quality scores and the snapshot engagement metrics recorded at the time of retrieval on either platform, suggesting that cross-sectional engagement measures may not directly reflect the objective quality of health information. These findings highlight the importance of evaluating video quality independently of engagement metrics and may inform future research on the role of platform characteristics in the dissemination of evidence-based health knowledge.

### 4.2. Practical significance

As 2 major video platforms, TikTok and Bilibili have played an important role in the dissemination of DN-related health information, with many included videos covering topics such as diagnosis and symptoms. The 2 platforms showed descriptive differences in observed engagement and content distribution.^[26]^ However, these implications should be interpreted cautiously in light of the descriptive and unadjusted nature of the present study. The limited emphasis on topics such as DN etiology, primary prevention, and lifestyle management suggests that important aspects of disease education may remain underrepresented on both platforms, which may reduce the completeness of information available to users.^[27]^ These findings may provide descriptive context for future efforts to improve the balance, completeness, and transparency of DN-related health communication on video platforms, particularly with regard to preventive information.^[28]^ In addition, different video formats may serve complementary roles, with shorter videos potentially attracting initial attention and longer videos potentially allowing more detailed explanations. Prior research has suggested that platform characteristics and content length can influence patterns of health information access.^[29]^ Future studies could explore whether factors such as source labeling and content presentation strategies are associated with improved understanding and trust in online health information.^[30]^

### 4.3. Limitations

This study has several limitations. First, social media engagement metrics are dynamic and may change substantially over time. In this study, likes, comments, shares, and favorites were recorded only at the time of retrieval and were therefore treated as cross-sectional snapshots rather than time-standardized indicators of audience response. Because upload dates and comparable time-adjusted engagement data were not consistently available across platforms, temporal standardization was not performed. Second, the observational and cross-sectional design precludes causal inference. Although Bonferroni correction was applied to reduce the risk of type I error from multiple comparisons, the analyses were based on unadjusted group comparisons rather than multivariable adjustment. Therefore, residual confounding cannot be excluded. Third, although established instruments were used to evaluate video quality, engagement metrics may be influenced by recommendation algorithms, differential exposure, and potential artificial traffic inflation. In addition, GQS, mDISCERN, and JAMA scoring still involve assessor judgment and may therefore retain some subjectivity. Finally, this study included only TikTok and Bilibili. Although both platforms are highly influential in China, other domestic and international social media platforms were not examined. Future research should incorporate broader cross-platform comparisons and, where possible, longitudinal, time-standardized, or multivariable analytical approaches.

## 5. Conclusion

In conclusion, DN-related videos on TikTok and Bilibili showed descriptive differences in observed engagement counts at the time of retrieval, whereas no statistically significant differences were observed in video duration or quality indicators after correction for multiple comparisons. In unadjusted descriptive comparisons, videos uploaded by experts were relatively shorter and showed more favorable results in selected quality-related measures, particularly GQS and JAMA, than videos uploaded by individual users. However, because this study was cross-sectional and unadjusted, these findings should be interpreted as exploratory descriptive associations rather than independent effects. Overall, the quality and reliability of DN-related video content remained variable across both platforms, highlighting the need for future longitudinal or multivariable studies.

## Acknowledgments

The authors express their gratitude to the Department of Nephrology, Shantou Traditional Chinese Medicine Hospital Affiliated to Guangzhou University of Chinese Medicine, for its support. This research received no external funding. The authors declare no conflicts of interest.

## Author contributions

**Conceptualization:** Min Wang, Baowen Gong.

**Data curation:** Min Wang, Xinyu Wang.

**Formal analysis:** Xinyu Wang.

**Investigation:** Xinyu Wang.

**Project administration:** Baowen Gong.

**Supervision:** Baowen Gong.

**Writing – original draft:** Min Wang.

**Writing – review & editing:** Baowen Gong.


